# Photodynamic Effects of *Thuja occidentalis* on Lung Cancer Cells

**DOI:** 10.3389/fphar.2022.928135

**Published:** 2022-07-15

**Authors:** Ayesha Loonat, Rahul Chandran, Janice Pellow, Heidi Abrahamse

**Affiliations:** ^1^ Laser Research Centre, Faculty of Health Sciences, University of Johannesburg, Johannesburg, South Africa; ^2^ Department of Complementary Medicine, Faculty of Health Sciences, University of Johannesburg, Johannesburg, South Africa

**Keywords:** lung cancer, photodynamic therapy, homeopathy, cytotoxicity, photosensitizer

## Abstract

The global incidence and mortality rates resulting from lung cancer encapsulate a need to identify more effective treatment protocols. Photodynamic therapy (PDT) and homeopathy offer possible anticancer therapies as part of a multi-disciplinary approach. Studies have identified the anticancer effects of *Thuja occidentalis* L. plant extracts. The aim of this study was to investigate the effects of *Thuja occidentalis* (TO) homeopathic mother tincture and TO mediated PDT (TO-PDT) on A549 lung cancer cells. Commercially available A549 cells were pre-treated with TO, or laser irradiation at 660 nm, or the combined treatment (TO-PDT). Cells were analyzed morphologically by inverted light microscopy and Hoechst stain; and biochemically by lactate dehydrogenase (LDH), adenosine triphosphate (ATP), and trypan blue assays. Cells treated with TO and TO-PDT demonstrated morphological changes in the cell and cell nuclei indicative of cell death. These groups exhibited a dose dependent increase in LDH release and a decrease in ATP levels and cell viability indicating its cytotoxic and antiproliferative potential. Furthermore, at the same doses, TO when photoactivated in PDT induced enhanced anticancer responses thereby surpassing the effects of treatment with the tincture alone. Results demonstrate how the direct cytotoxic effects of TO can be improved when administered as a photosensitizer in PDT to promote cancer cell death.

## 1 Introduction

With current socio-economic developments and epidemiological transitions, there is an increase in the distribution and prevalence of the main risk factors that can initiate lung cancer. This is reflected by the continuous rise in current and expected cases of lung cancer worldwide. Unsurprisingly, patients with lung cancer also have the highest mortality rates (Bray et al., 2018). Treatment of lung cancer relies on the combined administration of surgery, radiotherapy, and chemotherapy. Despite the improvements and implementation of these therapies, it often struggles to sustain patients’ expectations and treatment outcomes due to limitations of these therapies in the treatment of lung cancer. This includes lack of targeting precision, development of multi-drug resistance, and significant adverse events. These obstacles can be both a cause and a risk factor contributing to therapeutic failure and increased mortality rates (Mottaghitalab et al., 2019). Therefore, there is an expectation to continue to focus research efforts aimed at meeting crucial therapeutic demands based on highly effective treatment protocols with minimal adverse effects and long-lasting responses.

Integrative oncology embraces a multidisciplinary approach to the treatment regimen of cancer patients by administering both conventional and complementary medicines (CM) (Witt et al., 2017). The World Health Organization (WHO) defines CM as a diverse group of health care practices that do not form a part of the country’s own traditional or conventional therapies and are not fully integrated into the dominant healthcare system (Global report on traditional and, 1066). This combined approach has the potential to overcome critical challenges facing current conventional cancer therapies. This will, in turn, contribute towards improving patients’ quality of life (QoL) and promoting survival (Witt et al., 2017). Homeopathy is one such CM, that is, commonly used in parallel with other conventional therapies to aid in the supportive treatment of both oncological and non-oncological diseases as part of an integrative approach (Dehghan et al., 2020). Plant-based homeopathic mother tinctures (Ø) are hydroethanolic extractions of the entire plant or parts of the plant, prepared following specific guidelines in the homeopathic pharmacopeia (Das, 2019). Different from herbal tinctures which are far more concentrated in their dilution at 1:2 or 1:1 (Romm et al., 2010); Ø’s are prepared in a 1:10 dilution ratio (Steven, 2013).

Photodynamic therapy (PDT) is a photochemical process that involves the activation of a photosensitizer (PS) with an appropriate wavelength of light to produce cytotoxic reactive oxygen species (ROS) capable of initiating cell death (Chen et al., 2020). The efficacy of PDT lies in its multifunctional ability to elicit targeted cancer cell death with minimal invasiveness and drug resistance (Fan et al., 2019; Pinto da Silva et al., 2019). Successful PDT outcomes are highly dependent on selecting an appropriate PS. Currently, only a limited number of PSs are medically approved, most of which are commercially available in small amounts, and in many instances, only a few of them benefit patients entirely in clinical practice. In addition, the practical application of commonly used photophrin related PSs is limited by their slow elimination from the body, consequently resulting in adverse effects such as skin photosensitivity which may persist for weeks after the commencement of the treatment (Shi et al., 2019; Hu et al., 2020).

Natural agents are gaining much interest to determine alternative options and facilitate design strategies focused on improving safety, outcomes, and enhancing the efficacy of cancer treatments including PDT. Plant extracts contain a diverse group of bioactive compounds with anticancer capacity. These extracts can target multiple deregulated cellular pathways to induce cell death in an efficacious and selective manner and with minimal drug resistance and toxicity on healthy cells (Millimouno et al., 2014). Furthermore, these products are commercially available, environmentally sustainable, and cost-effective (Mansoori et al., 2019). Research data has provided evidence that demonstrates the presence of chromophores in various phytochemicals of plant extracts including furanocoumarins, polyacetylenic molecules, thiophenes, curcumins, xanthanoids, alkaloids, and antraquininones. These phytochemicals demonstrated photosensitizing capabilities by absorbing light at various wave-lengths within a phototherapy setting (Siewert and Stuppner, 2019). A review by Dong et al. (2021), demonstrated how hypericin-mediated PDT inhibited cell proliferation in various cancer models including colon, breast, glioma, cervical and hepatic tumour cells. Furthermore, the co-administration of hypericin-PDT and chemotherapeutic agents or other targeted therapies were able to increase the inhibitory effects of cell growth on cancer cells through the modulation of various proteins and genes (Dong et al., 2021). In an *in vitro* study on renal carcinoma cells, berberine was shown to be an effective PS in PDT (Lopes et al., 2019). The *in vitro* and *in vivo* effects of 5-Aminolevulinic acid (ALA) mediated PDT was shown to induce cell death in human colon cancer cells. In another study, ALA-PDT showed selected efficacy against aggressive adult T-cell leukemia without influencing normal lymphocytes (Sando et al., 2020). Curcumin has been extensively researched for its potential as a PS, and has demonstrated strong photocytotoxic effects in micromolar concentrations against a variety of cell lines. Furanocoumarins when photoactivated have been shown to be effective against various cancer cells through the inhibition of several pathways that play a key role in tumour development, and in the activation of proteins responsible for apoptotic induction (Kubrak et al., 2022).

*Thuja occidentalis* L. (TO) is a coniferous plant belonging to the Cupressaceae family with pro-apoptotic, anticarcinogenic, antiproliferative, antioxidant, and radio-protective properties. It is commonly known as the white cedar or arbor vitae and its name is Latin for tree of life (Silva et al., 2017). TO is indigenous to the north-eastern mixed and boreal forests of North America, and is grown as an ornamental tree in Europe (Danneyrolles et al., 2017). It is an important Native American ceremonial plant which has been widely utilized in ethnomedicine. The plant is greatly honoured by the Ojibwa culture by the name Nookomis Giizhik, meaning Grandmother Cedar who used the soft twigs to make soups and teas for the treatment of various ailments (Pudełek et al., 2019). In traditional medicine, TO has been used to treat diseases of the respiratory system (bronchial catarrh), urinary and reproductive systems (enuresis, cystitis, amenorrhoea), as well as rheumatic and autoimmune diseases (Stan et al., 2019). In a homeopathic clinical setting, TO mother tincture is prescribed in the treatment regimen of pathological growths such as tumours, and acute and chronic respiratory tract infections. Cell death owing to TO in cancer cells can be attributed to its primary active phytochemical thujone and to a lesser extent its polysaccharide and flavonoid fractions (Alves et al., 2014). Thujone is associated with the activation of BAX, cytochrome c, and caspase 3; all in favor of pro-apoptotic signaling (Ijaz et al., 2018). A study by Siveen and Kuttan (2011) demonstrated the immunostimulatory activity of thujone on the humoral and cell-mediated immune system during solid tumour development. The augmentation of the immune system by thujone increased natural killer (NK) cell activity, antibody-dependent cellular cytotoxicity (ADCC), and cytotoxic T lymphocyte generation (Siveen and Kuttan, 2011). Other studies have shown how thujone can exert pro-apoptotic and anti-invasive effects. The attenuating effect of thujone was further supported with the accumulation of ROS (Ijaz et al., 2018; Pudełek et al., 2019). An *in vivo* study by Sunila et al. (2011) showed the effects of TO and its polysaccharide on tumour-bearing mice which effectively stimulated cell-mediated immunity through the enhancement of NK activity, ADCC, and antibody-dependent complement-mediated cytotoxicity. Furthermore, it also showed a decrease in pro-inflammatory cytokines, as a result inhibiting metastasis of tumour cells (Sunila et al., 2011). Mukherjee et al. (2014) demonstrated the target specific and chemopreventative role of flavonol isolated from the ethanolic leaf extracts of TO on A549 cells *in vitro* and Swiss albino mice *in vivo*. Findings demonstrated the upregulation of p53 after exposure to flavanol *in vitro* and resulted in the inhibition of cellular proliferation and tumour growth *in vivo*. Moreover, the flavonol at its specific dose was neither cytotoxic to normal L-132 lung cells *in vitro*, nor was able to raise any toxicity in mouse bodies, *in vivo* (Mukherjee et al., 2014).

To expand the anticancer potential of TO, this project aimed to be a starting point towards discovering the effects of TO mediated PDT (TO-PDT) to observe the potential synergistic benefits in enhancing cancer cell death, which will allow for further research in the field. Additionally, this study was the first to introduce the role of TO as a possible natural PS in PDT applications. Results demonstrate how the direct cytotoxic effects of TO can act synergistically with the photochemical reactions produced during PDT to promote cancer cell death, which could prove to be a promising lung cancer treatment strategy.

## 2 Materials and Methods

### 2.1 Cell Culture

A549 lung cancer cells (ATCC CCL-185, P-17) were cultured in Roswell Park Memorial Institute 1640 Medium (RPMI, Sigma-Aldrich, R8758) supplemented with 10% foetal bovine serum (FBS, Gibco, 306-00301), 1% antibacterial (Penicillin-streptomycin, Sigma-Aldrich, P4333) and 1% antifungal (Amphotericin-B, Sigma-Aldrich, A2942) agents. Cells from passage 17 were used and taken for experimentation every four to five passages. It was made sure that all the experimental groups include cells from same passage number. Approximately 5 × 10^5^ cells were seeded in 3.4 cm diameter culture plates. All cultures were maintained at 37°C with 5% carbon dioxide (CO_2_) and 85% humidity for 24 h to allow the cells to attach. Culture plates with more than 90% confluence were used for further experiments.

### 2.2 *Thuja occidentalis*

The homeopathic mother tincture was bought from a reputable and registered homeopathic company (Fusion Homeopathics, South Africa). This company imports its mother tinctures from Gehrlicher Pharmaceutical Extracts (GmbH) (manufactured according to the appropriate monograph rule 3a of the German and European Homeopathic Pharmacopoeia). The tincture was stored at room temperature, away from direct sunlight. To assess for optimal dosing parameters and to identify relevant treatment-related toxicities, different doses of TO were added to culture plates for 24 h before carrying out cytotoxicity assays. TO at 5, 10, and 15 μl were selected as the final doses for further experiments.

#### 2.2.1 Ethanol Cytotoxicity Study

TO is prepared using a 61% ethanol solvent. To keep the concentration of the solvent in the tincture at the most suitable doses for biological experimentation, an ethanol group was included in a preliminary study as a tincture solvent control. Cells received predetermined doses of 61% ethanol (identical to the homeopathic tincture but without the active ingredients) for 24 h. An untreated group receiving neither any tincture nor ethanol was included as a control. Both groups were subjected to morphological and biochemical studies including inverted light microscopy and trypan blue staining. As the ethanol treated cells had no significant change to the untreated control at doses of 5, 10, and 15 μl, the group was not included in further experiments.

### 2.3 Laser Set-Up and Parameters

For treatment regimens with light, cell samples were irradiated using a diode laser supplied by the Council for Scientific and Industrial Research (CSIR), National Laser Centre (NLC) of South Africa, at a wavelength of 660 nm and a 5 J/cm^2^ fluence rate. A power meter (FieldMate) was used to determine the power output of the laser at bench level in the dark. To obtain a fluency of 5 J/cm^2^, the duration of laser irradiation was calculated using the following formulae:

Fluency (J/cm^2^) = time (s) × [power output (W) ⁄ Surface area (cm^2^)].

Culture plates were protected from extraneous light sources, and one culture dish was illuminated at a time. The culture dishes requiring irradiation were placed directly under the laser beam with their lids removed. All control and experimental culture plates were covered in foil and placed back in the incubator for 24 h before carrying out post-irradiation assays**.** Irradiation parameters can be found in Table 1.

**TABLE 1:** Laser irradiation parameters.

Name and type	Diode laser
Spectrum	660 nm
Wave emission	Continuous wave
Spot size	9.1 cm^2^
Power output	87.6 mW
Power density	9.3 mW/cm^2^
Fluency	5 J/cm^2^
Irradiation time	9 min, 3 s (s)

### 2.4 Experimental Parameters

Culture plates were incubated for 24 h with media containing TO at concentrations of 5 μl, 10 μl, and 15 μl; irradiation alone at 660 nm (5 J/cm^2^ fluence rate); or the combined treatment (5 µl TO-PDT, 10 µl TO-PDT, and 15 µl TO-PDT). An untreated control was included as a means of comparing treatment responses. All treated and untreated groups were analyzed morphologically by inverted light microscopy and the Hoechst stain assay; as well as biochemically by LDH, ATP, and the trypan blue assays.

#### 2.4.1 Cellular Morphology- Inverted Light Microscopy

The morphology and cellular density of the treated and untreated cells were analyzed using an inverted light microscope (Wirsam, Olympus CKX41). The microscope is connected to a camera to allow for the live visualization of cells. Culture plates were placed individually under the microscope with their lids removed in a darkened room. Images were captured on a computer using the GetIT analysis program.

#### 2.4.2 Nuclear Morphology- Hoechst Stain Assay

Any alterations in the nuclear morphology of the treated and untreated cells were assessed by the Hoechst stain assay. Cells were seeded in 3.4 cm diameter culture dishes over sterile coverslips and allowed to reach above 80% confluence. Once confluent, cells were exposed to the different treatments. After 24 h of incubation at 37°C with 5% CO_2_ and 85% humidity, each coverslip was stained with 200 μl of the Hoechst working stock solution (Hoechst 33258, Invitrogen, H1398) and incubated at room temperature for 30 min. The solution was removed from each coverslip, and cells were rinsed with phosphate-buffered saline (PBS, Sigma-Aldrich, R8758) to remove any remaining stain. Fluorescence was observed in a dark room with a fluorescent microscope (Axio observer Z1, Carl Zeiss) using a broadband 4′,6-Diamidino-2-Phenylindole (DAPI) filter set, measured at an excitation wavelength of 352 nm and an emission wavelength of 455 nm.

#### 2.4.3 Cytotoxicity-Lactate Dehydrogenase Assay

The membrane integrity of cells was assessed by estimating the amount of LDH pre-sent in the culture media. The cytosolic enzyme LDH is released into the media due to cell membrane damage. The CytoTox 96^®^ Non-Radioactive Cytotoxicity Assay (Anatech Promega, G400) is a quantitative method used to measure the LDH released in all control and experimental groups. Equal volumes (50 μl) of reconstituted LDH reagent and cell culture medium were micro pipetted in a clear bottom 96 well plate. The entire 96 well plate was covered with tin foil, mixed, and incubated in the dark at room temperature for 15 min. The colorimetric mixture was measured spectrophotometrically at 490 nm (Perkin–Elmer, Separation Scientific, VICTOR3™).

#### 2.4.4 Cellular Proliferation- Adenosine Triphosphate Luminescent Assay

The ATP Cell Titer-Glo^®^ luminescent assay (Anatech Promega, G7570) is a homogeneous method for the determination of cellular proliferation and quantification of ATP present in metabolically active cells. Equal volumes (50 μl) of reconstituted ATP reagent and the cell suspension were micro pipetted into an opaque-walled luminescent 96 well plate (white). The entire plate was covered with foil and the cells and reagent mixture were mixed on a shaker for 5 min to induce cell lysis. The cells were incubated in the dark at room temperature for 30 min to stabilize the luminescent signal. The luminescent signal was read using the 1420 multilabel counter victor3 (Perkin-Elmer, Separation Scientific, VIC-TOR3™) in relative light units (RLU).

#### 2.4.5 Cellular Viability- Trypan Blue Assay

Cell viability in all groups was quantified using the trypan blue dye exclusion assay. Trypan blue (Sigma-Aldrich, T8154) was used to stain the cells by diluting 10 μl of the cell suspension into 10 μl of 0.4% trypan blue dye. A volume of 10 μl of stained cells was loaded into each side of a plastic, disposable cell counting chamber slide. Cells with damaged membranes (non-viable) will take in the blue chromophore dye, whereas the cells with intact cell membranes (viable) will not absorb the dye and hence, remain clear. An automated cell counter (CountessTM Automated Cell Counter, Thermo Fischer, AMQAX1000) was used for the cell count and viability estimation.

### 2.5 Statistical Analysis

Data were obtained from representative independent experiments and expressed as the mean ± standard error (SE). Experiments were repeated in triplicate, and assays were run in duplicate (*n* = 6). The SPSS Data Analysis Software was used to collect and process the results. Data were statistically analyzed using the one-way ANOVA and Dennett’s multiple comparison test. Statistical significance was set at *p < 0.05 when compared to the control.

## 3 Results

### 3.1 Ethanol Cytotoxicity Study on A549 Cells

To investigate the cytotoxic effects of ethanol, A549 cells were pre-treated with 61% ethanol for 24 h at different doses and compared to untreated control cells. The cytotoxic and antiproliferative effects were observed morphologically by an inverted light microscope and biochemically by trypan blue staining.

#### 3.2.1 Cellular Morphology by Inverted Light Microscopy

**FIGURE 1:**
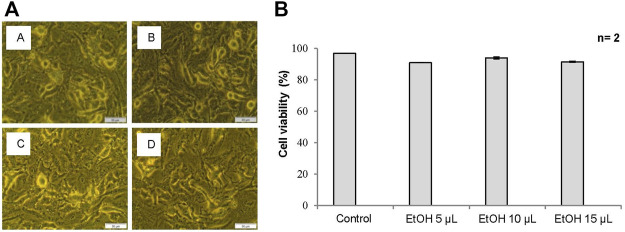
**(A)** Cell morphology of A549 cells after exposure to different doses of 61% ethanol (EtOH) under an inverted light microscope (x200 Magnification). Figure 1A Untreated control, **(B)** 5 µl EtOH, **(C)** 10 µl EtOH, and **(D)** 15 µl EtOH. Scale bar 50 µm. **(B)** The percentage (%) of viable cells using trypan blue assay in A549 cells treated with 61% EtOH. Data is represented as the mean standard error (±SE).

An inverted light microscope was used to assess any morphological changes in the cell and cell density of A549 cells treated with 61% ethanol at the selected doses that may be indicative of apoptosis and hence cell death. Cells treated with ethanol at doses up to 15 µl demonstrated no significant change in cell morphology and density compared to the control. Cells at these doses remained attached to their respective culture plates at high densities and retained their normal morphological shape. Figure 1A demonstrates the morphological changes in A549 cells treated with 61% ethanol at different doses compared to untreated control cells.

#### 3.2.2 Cellular Viability by Trypan Blue Assay

The trypan blue assay was used to assess the viability of A549 cells treated with 61% ethanol at different doses. Cells treated with ethanol at doses up to 15 µl demonstrated no significant change in cell viability when compared to the control and is thus associated with the absence of cytotoxicity. Figure 1B shows the viability of A549 cells treated with 61% ethanol at different doses compared to the untreated control.

### 3.3 Experimental Study

To investigate the cytotoxic and antiproliferative effects of the treatment, A549 cells were pre-treated with TO, TO-PDT, and irradiation alone for 24 h and compared to untreated control cells. TO was delivered to cells in doses of 5, 10, and 15 µl. A 660 nm diode laser was used to deliver treatment regimens with light at a fluence rate of 5 J/cm^2^. To obtain this fluency, the duration of laser irradiation was calculated using the power output from the 660 nm laser. The power output for the 660 nm laser was 87.6 mW. Therefore, the time calculated to deliver a fluence of 5 J/cm^2^ to a culture plate requiring irradiation was 9 min and 3 s. The cytotoxic and antiproliferative effects were observed morphologically and biochemically using various assays. Statistically significant at *p < 0.05, **p < 0.01, and ***p < 0.001.

#### 3.3.1 Cellular Morphology by Inverted Light Microscopy

**FIGURE 2:**
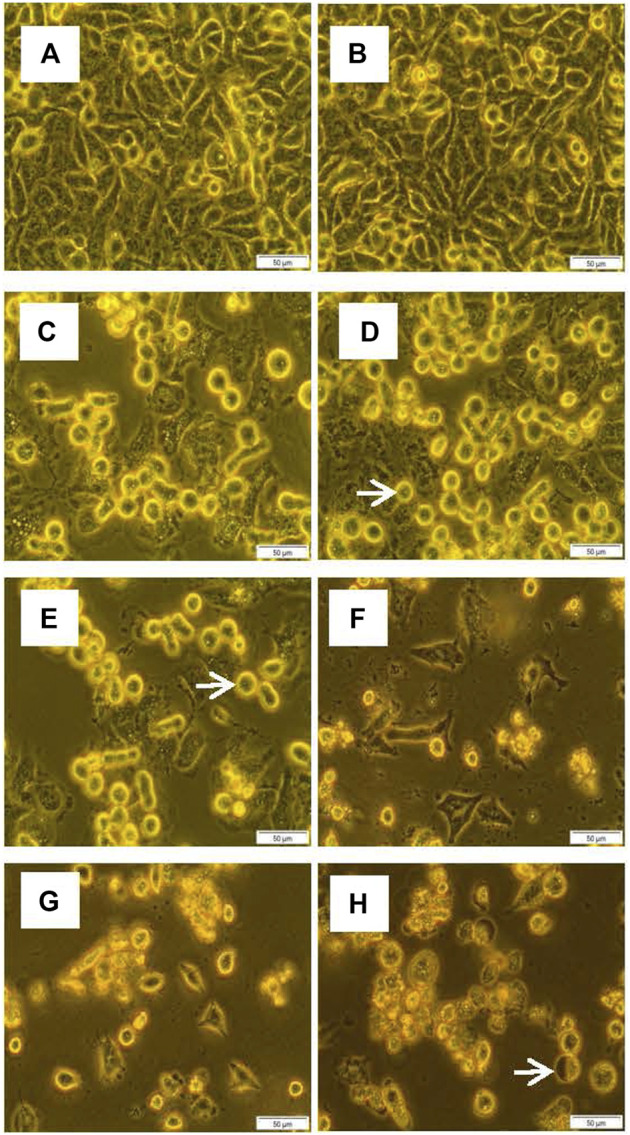
Cell morphology of A549 cells after exposure to different doses of *Thuja occidentalis* homeopathic mother tincture (TO) and TO mediated photodynamic therapy (TO-PDT) under an inverted light microscope (x200 Magnification). Figure 3 **(A)** Untreated control, **(B)** Irradiation only, **(C)** 5 µl TO, **(D)** 10 µl TO, **(E)** 15 µl TO, **(F)** 5 µl TO-PDT, **(G)** 10 µl TO-PDT, **(H)** 15 µl TO-PDT. Scale bar 50 µm. White arrows indicate examples of rounded up dead cells.

Cells in all control and experimental groups were observed under an inverted light microscope to assess any changes in the cell that may be attributed to cell death. Cells treated with TO and TO-PDT demonstrated significant morphological changes when compared to the control. Cells in the control group retained their normal shape and remained attached to their respective culture plates in high densities. Cell detachment, rounding up of cells, and cell debris were observed in cells treated with TO and TO-PDT, with increasing evidence of cell death in cells treated with 15 µl of TO-PDT. Cells treated with irradiation alone demonstrated no observable changes in cell morphology when compared to the control. Figure 2 demonstrates the morphology of the A549 cells in the control and experimental cell groups.

#### 3.3.2 Nuclear Morphology by Hoechst Stain Assay

**FIGURE 3:**
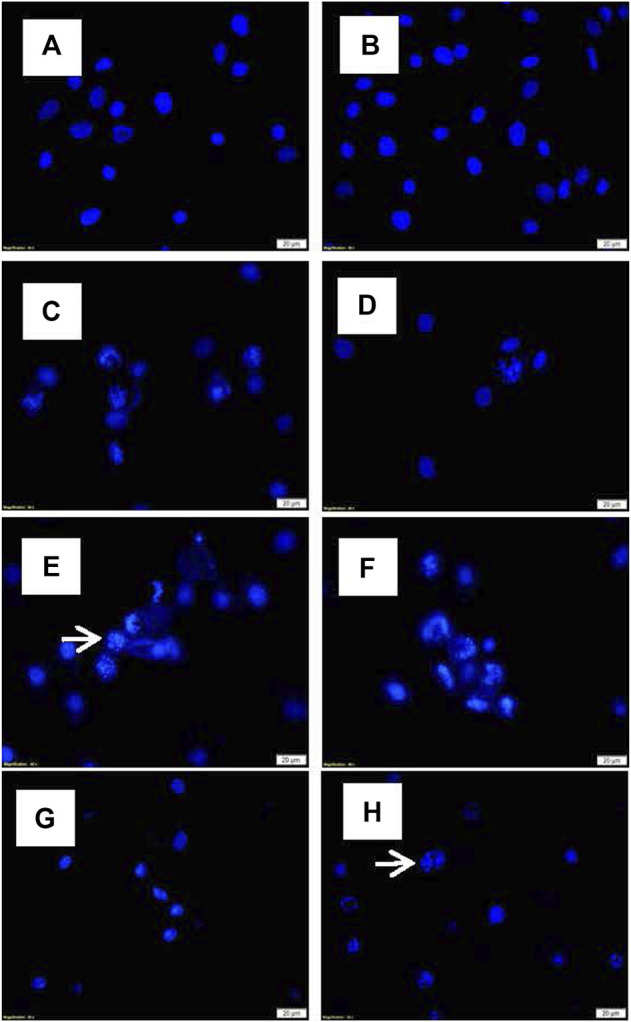
Nuclei morphology of A549 cells after exposure to different doses of TO and TO-PDT under a fluorescent microscope (x400 Magnification). **(A)** Untreated control, **(B)** Irradiation only, **(C)** 5 µl TO, **(D)** 10 µl TO, **(E)** 15 µl TO, **(F)** 5 µl TO-PDT, **(G)** 10 µl TO-PDT, **(H)** 15 µl TO-PDT. Scale bar 20 µm. White arrows indicate examples of abnormal cell nuclei.

Alterations in the nuclear morphology of the treated cells were explored using the Hoechst fluorescent stain assay and compared to the control cells. Changes in the nuclei morphology associated with apoptosis; including abnormally shaped nuclei, nuclei shrinkage, chromatin condensation, and nuclei fragmentation were observed in cells treated with TO and TO-PDT. Cells treated with irradiation alone demonstrated no observable changes in the cell nuclei when compared to the control. Figure 3 shows the morphology of the cell nuclei in all experimental and control groups.

#### 3.3.3 Cytotoxicity by Lactate Dehydrogenase Assay

**FIGURE 4:**
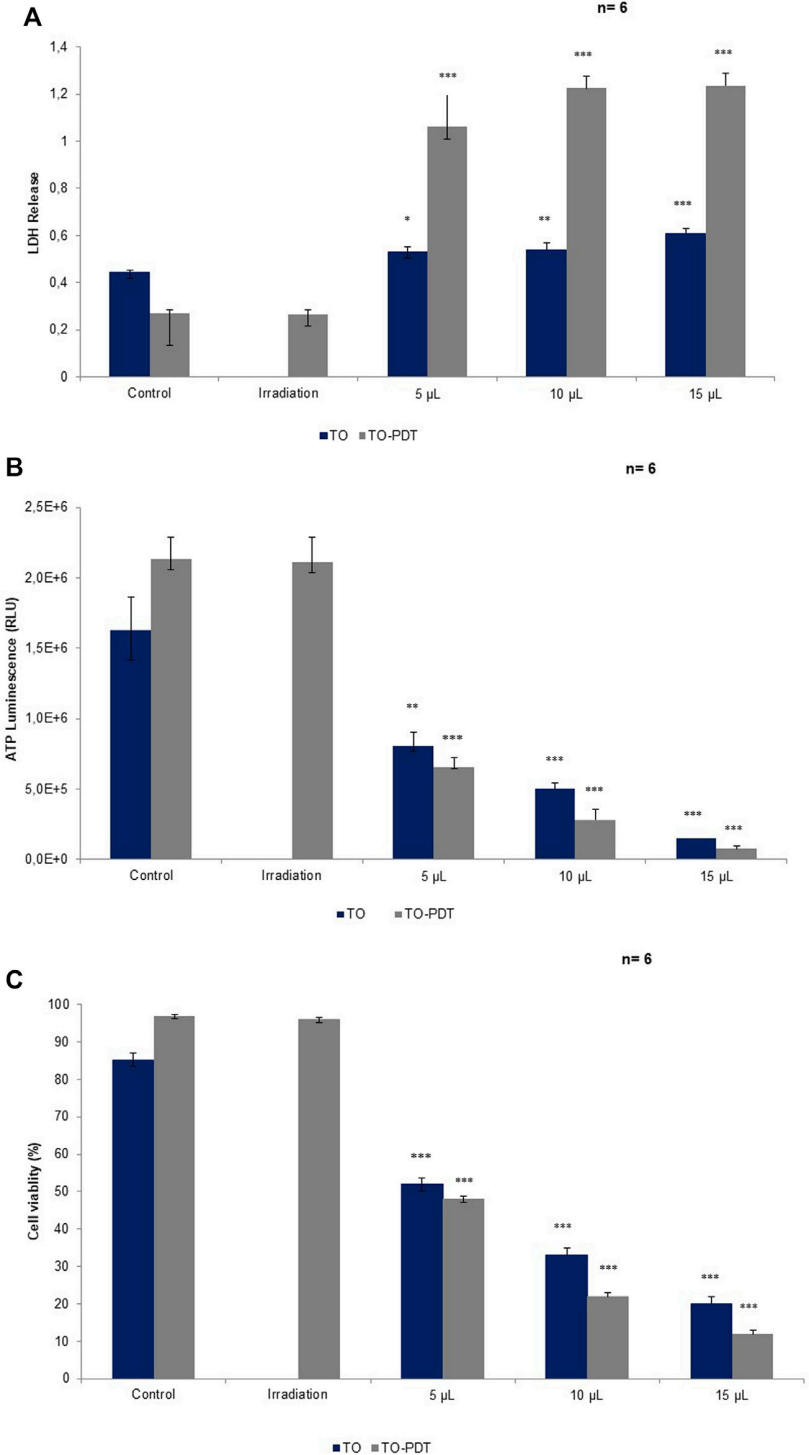
A comparison of **(A)** LDH, **(B)** ATP, and **(C)** cell viability levels on A549 cells treated with *Thuja occidentalis* homeopathic mother tincture (TO) and TO mediated photodynamic therapy (TO-PDT) at the same doses of the tincture. At the same doses, TO when used as a photosensitizer in PDT, produced amplified antiproliferative and cytotoxic responses on A549 cells compared to cells treated with TO alone.

The lactate dehydrogenase (LDH) CytoTox96^®^ colorimetric assay was used to investigate the *in vitro* cytotoxic effects of the different treatments on A549 cells compared to the control cells. Cell death is accompanied by a loss of cell membrane integrity which allows the intracellular LDH to release into the extracellular media. The extracellular LDH was quantified 24 h post-treatment. Cells treated with TO at 5 (*p* < 0.05), 10 (*p* < 0.01), and 15 (*p* < 0.001); and TO-PDT (*p* < 0.05) demonstrated significant dose-dependent decreases in LDH levels when compared to the control. The highest cytotoxic effects were observed in A549 cells after using a dose of 15 μl TO-PDT. Cells treated with irradiation alone demonstrated no significant change in comparison to the control. Figure 4A demonstrates the levels of LDH in all experimental and control groups.

#### 3.3.4 Cellular Proliferation by Adenosine Triphosphate Luminescent Assay

The adenosine triphosphate (ATP) Cell Titer-Glo^®^ luminescent assay was used to determine the antiproliferative potential of the different treatment groups on A549 cells when compared to the control. Metabolically active cells are associated with higher levels of ATP due to the increased proliferation of cells compared to metabolically inactive cells. Cells treated with TO at 5 (*p* < 0.01), 10, and 15 (*p* < 0.001); and TO-PDT (*p* < 0.001) demonstrated significant dose-dependent decreases in the levels of intracellular ATP when compared to the control. Results demonstrate the antiproliferative potential of both therapies against the A549 cells. Cells treated with irradiation alone, demonstrated no significant change in ATP levels when compared to the control. Figure 4B shows the levels of ATP in all experimental and control groups.

#### 3.3.5 Cellular Viability by Trypan Blue Assay

The trypan blue assay was used to investigate the viability of cells in all experimental and control groups. A decrease in cell viability is directly associated with cell death and vice versa. Cells treated with TO (*p* < 0.001) and TO-PDT (*p* < 0.001) demonstrated a dose-dependent decrease in the viability of the cells when compared to the control. Cells treated with irradiation alone, demonstrated no significant change in the viability of cells when compared to the control and both maintained an average viability rate above 90%. Figure 4C shows the viability of cells in all experimental and control groups.

## 4 Discussion

Lung cancer is at the forefront of cancer-related mortality worldwide, and despite advances in current treatments it still has a 5-year survival rate estimated at 15% of all stages combined (Duruisseaux and Esteller, 2018). This highlights the need for further investigations that may identify alternative treatment pathways that provide more efficient solutions for diverse situations. This study introduced and proposed the combinatory role of PDT and homeopathy in an attempt to provide new perspectives in the search for optimal lung cancer therapeutics. Studies carried out *in vivo* and *in vitro* have demonstrated and maintained the role of TO as an effective anticancer agent that can act as a natural obstacle in limiting cancer cell growth and progression. To expand the cytotoxic potential of TO, this study investigated the effects of TO homeopathic mother tincture when used as a PS in PDT on A549 lung cancer cells to observe any additive value of the combined treatment in enhancing cancer cell death.

Ethanol above its toxic threshold can alter the cellular environment and cause disruptions in the physical activities of cells. Thus, ethanol at toxic dosages can promote cell death which can consequently alter experimental outcomes and results (Timm et al., 2013). TO is pre-pared in a 61% hydroethanolic solvent; therefore to attribute the anticancer effects of the treatment to its plant actives, the solvent at its selected dose had to be biocompatible and non-toxic to cells. Both the morphological and viability studies demonstrated that ethanol in doses up to 15 µl had no significant observable differences when compared to the untreated control. This would imply that 61% ethanol in optimized doses (5, 10, and 15 µl) had no direct cytotoxic effects on A549 cells, and any signs of cell death can be attributed to TO.

Cells treated with TO and TO-PDT displayed significant morphological changes under an inverted light microscope which provided preliminary evidence of cell death. This included loss of cell-to-cell contact, cell shrinkage, reduced cellular density, rounding up of cells, and cell detachment from their respective culture plates; all associated with features indicative of apoptosis and hence, cell death. Results are consistent with nuclear morphology studies, which demonstrate the ability of the treatments in inducing DNA damage. On the Hoechst stain, cells treated with TO and TO-PDT displayed irregularly shaped nuclei, chromatin condensation, nuclear fragmentation, and shrinkage; all of which are associated with the typical features of apoptosis and negative consequences for cell survival and progression. Cells treated with irradiation alone were unable to elicit any significant change morphologically or biochemically compared to the control cells and are hence, associated with the absence of any positive anticancer effects. Results from the morphological studies correlate with results obtained from biochemical parameters. Cells treated with TO and TO-PDT demonstrated a statistically significant dose-dependent increase in LDH levels. The LDH results are complemented by the dose-dependent decrease in ATP and cell viability, which confirms the antiproliferative and cytotoxic potential of the therapies. The decline in ATP is associated with a decrease in the metabolic activity of the cells meaning that the cells can no longer sustain growth signaling and cell proliferation. ATP deprivation is a common feature occurring in apoptosis, necrosis, and autophagic cell death mechanisms (Nikoletopoulou et al., 2013; Kim, 2018).

These results validate previous research studies that elucidate and demonstrate the independent ability of TO herbal and homeopathic extracts in inducing cancer cell death. In the *in vitro* study by Biswas et al., TO homeopathic mother tincture was shown to decrease cell viability and proliferation and induce cell death through apoptotic induction in A375 cancer cells by favouring mitochondrial transmembrane collapse, internucleosomal DNA fragmentation, increased ROS generation, and the activation of caspase-3 (Biswas et al., 2011). Torres et al. (2016) provided a detailed mechanism of the pro-apoptotic and anti-angiogenic properties of the thujone fractions isolated from TO extracts on glioblastoma cells *in vitro*. The thujone components induced their anticancer effects through the inhibition of angiogenic factor production, decrease in the tube formation of endothelial cells, and decreased expressions of vascular endothelial growth factors (VEGF) and angiopoietin-4. These factors are essential for tumour growth and survival in a hypoxic environment (Torres et al., 2016). In an *in vitro* study by Saha et al. (2014), TO extracts were shown to induce apoptosis in a ROS-P53 feedback loop on breast cancer cells (Saha et al., 2014). These studies showed the preferential induction of the cytotoxic effects on cancer cells while maintaining the intact structure and integrity of healthy cells.

TO when photoactivated, revealed higher anticancer activity levels on A549 cells compared to cells treated with TO only (Figure 4). At the same doses, cells treated with TO-PDT had the ability to remarkably enhance the cytotoxic and antiproliferative potential in A549 lung cancer cells 24 h post-irradiation by favouring higher LDH release and lower ATP production and cell viability, thereby surpassing the effects of treatment with TO alone. This indicates how the direct cytotoxic effects of TO can act synergistically with the photochemical reactions produced during PDT to promote cell death. This implies that TO has a positive absorption wavelength at the red region specifically at 660 nm, which falls within the therapeutic window of biological tissue where increased transmission and high ROS production are at its maximum (Donohoe et al., 2019). Cell death after PDT is mediated predominantly through apoptosis which is associated with irreversible cell death (Villacorta et al., 2017). According to phytochemical studies, TO based preparations contain various active constituents including essentials oils, coumarins, flavonoids, tannins and proanthocyanidines (Caruntu et al., 2020). In a PDT setting, coumarins and its derivatives play an active role in research as a naturally occurring organic PS due its strong photophysical and photochemical properties (Köksoy et al., 2019). A study by Gangopadhyay et al. (2015), revealed the photodynamic effects of a titanium dioxide-coumarin nanoconjugate on MDA-MB-231 breast cancer cells. Coumarin derivatives were selected as the chromophore in the complex due to its photophysiochemical abilities (Gangopadhyay et al., 2015). In TO, coumarins are present in the form of p-coumaric acid (PCA), and umbelliferone (7-hydroxycoumarin) (Caruntu et al., 2020). PCA is a phenolic compound with antioxidant, anticancer and anti-inflammatory properties. Kianmehr et al. (2020) demonstrated the anticancer activity of PCA and low-level irradiation against human malignant melanoma cells. The combined treatment was able to decrease cell viability, possibly through apoptotic pathways (Kianmehr et al., 2020). 7-hydroxycoumarins have been shown to exhibit anticancer, anti-inflammatory, and antimutagenic properties. These agents are considered to be common precursors of furanocoumarin derivatives depending on the prenylation position of the 7-hydroxycoumarin. At the C6 position, 7-hydroxycoumarin gives rise to the psoralen derivative and at the C8 position it gives rise to the angelicin derivative (Karamat et al., 2014). Further studies should aim to discover the exact phytochemicals that could be responsible for its interactions and contributions in a PDT setting.

While TO-PDT may be an effective treatment strategy against lung cancer, further and more complex investigations are needed to determine the mechanism of action of the therapy and to establish optimal dosimetric, treatment, and safety parameters that would allow for wider-ranging interpretations of the current findings.

## 5 Conclusion

This study has corroborated previous research that demonstrates the cytotoxic and antiproliferative properties of TO and has implicated its efficacy as a potential anticancer agent. This study was also the first to introduce the role of TO as a possible natural PS in PDT applications, which by this study has been revealed to be far more effective in inducing cancer cell death than that produced by TO independently. A549 cells treated with TO and TO-PDT showed a dose-dependent increase in LDH with a concomitant decrease in ATP and cell viability levels that were statistically significant and biologically plausible. Furthermore, changes in the cell and cell nuclei indicative of apoptosis were observed under morphological analyses. The implementation of TO in PDT applications presents a possible anticancer strategy in the treatment of lung cancer. The experiment can be replicated on different types of cell lines to determine the efficacy of TO and TO-PDT on other types of cancer. However, an extensive amount of research is encouraged and needed to validate the laboratory findings in both *in vitro* and *in vivo* studies before treatment regimens with TO-PDT become discernible. Further research will aim to compare the photodynamic effects of TO to other medically approved PSs, to determine if TO-PDT has any additional and synergistic benefits aimed at overcoming the limitations of current conventional PSs.

## Data Availability

The original contributions presented in the study are included in the article/supplementary materials, further inquiries can be directed to the corresponding author.
